# Methods for Mapping Neuronal Activity to Synaptic Connectivity: Lessons From Larval Zebrafish

**DOI:** 10.3389/fncir.2018.00089

**Published:** 2018-10-25

**Authors:** Adrian A. Wanner, Ashwin Vishwanathan

**Affiliations:** Princeton Neuroscience Institute, Princeton University, Princeton, NJ, United States

**Keywords:** zebrafish, connectome, olfactory bulb, hind brain neurons, electron microscopy, two-photon (2P), neural circuit

## Abstract

For a mechanistic understanding of neuronal circuits in the brain, a detailed description of information flow is necessary. Thereby it is crucial to link neuron function to the underlying circuit structure. Multiphoton calcium imaging is the standard technique to record the activity of hundreds of neurons simultaneously. Similarly, recent advances in high-throughput electron microscopy techniques allow for the reconstruction of synaptic resolution wiring diagrams. These two methods can be combined to study both function and structure in the same specimen. Due to its small size and optical transparency, the larval zebrafish brain is one of the very few vertebrate systems where both, activity and connectivity of all neurons from entire, anatomically defined brain regions, can be analyzed. Here, we describe different methods and the tools required for combining multiphoton microscopy with dense circuit reconstruction from electron microscopy stacks of entire brain regions in the larval zebrafish.

## 1. Introduction

The larval zebrafish has been gaining a lot of traction as a model system in systems neuroscience (Friedrich et al., 2010). From a model system point of view for neuroscience, the larval zebrafish sits in between the fly and the mouse, two of the most popular model systems. At larval stage, 4–7 days post fertilization (dpf), these fish have approximately 100,000 neurons in their nervous systems (Hill et al., 2003). The larvae are accessible to a variety of tools that include advanced genetic manipulation, high-throughput screening, behavioral assays, electrophysiology, and functional imaging. More importantly, at their larval stage they are optically transparent which makes them accessible for functional imaging and allows brain-wide monitoring of neuronal activity (Ahrens et al., 2013; Dal Maschio et al., 2017). To this end many studies use the larval zebrafish to study mechanisms by which activity in networks of neurons can lead to meaningful sensory processing and eventually behavior. In terms of behaviors the larvae display a rich set of behaviors like prey-capture, looming, and foraging that can be studied either in freely moving animals or in a virtual environment where typically the read out is in the form of eye and tail movements (Wyart et al., 2009; Ahrens et al., 2012; Bianco and Engert, 2015; Temizer et al., 2015; Dunn et al., 2016a,b; Naumann et al., 2016) and have thus been used extensively to investigate sensorimotor transformations (Mathuru et al., 2012; Barker and Baier, 2015).

From a systems neuroscience perspective, having access to the function, genetics, structure, and a wiring diagram of the neurons involved is key to understanding how fundamental computations are performed in the brain. Recent studies have shown how it is possible to extract single-cell transcriptomic data from entire brains or regions (Pandey et al., 2018; Raj et al., 2018). Similarly, it is also possible to functionally image the activity of the entire nervous system (Ahrens et al., 2013; Dal Maschio et al., 2017). However, detailed ultrastructural information on connectivity of the underlying neuronal circuits is still missing for most zebrafish brain regions. For a mechanistic understanding of neuronal computations and information flow it is essential to reconstruct the neuronal circuits at synaptic resolution. While low resolution electron microscopy (EM) and light microscopy is sufficient for mapping axonal projection patterns and somata locations (Randlett et al., 2015; Förster et al., 2017a; Hildebrand et al., 2017), a detailed mapping of neuronal circuits requires EM at synaptic resolution (Wanner et al., 2016b; Vishwanathan et al., 2017; Svara et al., 2018).

We present here a comprehensive step-by-step guide for neuronal circuit reconstruction in the larval zebrafish using serial block-face scanning electron microscopy (SBEM) or automated tape collecting ultra-microtome based scanning electron microscopy (ATUM-SEM). We outline the typical workflow for dense and targeted reconstruction of connectivity and activity of neurons using correlative light and electron microscopy. We exemplify the workflow by highlighting two recent studies in which significant parts of neuronal circuits have been reconstructed at synaptic resolution in the olfactory bulb and the hindbrain of the larval zebrafish, respectively (Table 1) (Wanner et al., 2016b; Vishwanathan et al., 2017). The main factors contributing to the quality of a volume EM stack are tissue preservation, voxel resolution, image contrast, and image registration. The most time consuming step and at the same time the major bottleneck in combining functional imaging and EM-based circuit analysis is the neuron reconstruction and synapse annotation. The accuracy and efficiency of image annotation depends highly on the quality of the underlying EM image stacks, thus it is crucial to optimize the EM preparation and image acquisition for the subsequent circuit reconstruction. Each of these steps takes significant time to optimize and to get right. By comparing the two methods that were used we hope to provide the reader a detailed overview of the methods and tools required for accomplishing such reconstructions.

**Table 1:** Dataset comparison.

	**Wanner et al**.	**Vishwanathan et al**.
1. Imaged region, dimensions	Olfactory bulb (OB) - 72×108×119 μm	Hindbrain (HB) - 120×250×80 μm
2. Imaging method	Scanning block face imaging (SBEM)	Automated tape collecting ultramicrotome (ATUM)
3. Imaging mode	Back scattered electrons	Back scattered electrons
4. Imaging resolution (lateral, axial)	9 × 9 × 25 nm	5 × 5 × 45 nm
5. Image alignment	Custom MATLAB tools	TrakEM2
6. Light and electron microscopy registration	Custom MATLAB tools	TrakEM2, MATLAB
7. Image segmentation	-	Deep nets (https://github.com/seung-lab)
8. Neuron reconstruction	Manual skeleton tracing and synapse annotation of ~98% of all neurons (*n* > 1,000) in the larval OB	Manual skeleton tracing and synapse annotation of 22 neurons, volumetric segmentation of ~2000 neurons in the HB
9. Software:	PyKNOSSOS (Wanner et al., 2016a) https://github.com/adwanner/PyKNOSSOS KNOSSOS (Helmstaedter et al., 2011) https://knossostool.org	TrackEM2 (Cardona et al., 2012) https://imagej.net/TrakEM2 BigWarp http://imagej.net/BigWarp

## 2. Functional imaging

The recent advances in optogenetic tools and light microscopy (LM) have revolutionized population scale imaging of neuronal activity at cellular resolution. The advent of better transgenic tools (Halpern et al., 2008; Kimura et al., 2014; Marquart et al., 2015; Förster et al., 2017b), calcium reporters (Chen et al., 2013; Piatkevich et al., 2018), imaging techniques like light sheet microscopy (Ahrens et al., 2013; Panier et al., 2013) and two photon microscopy (O'Malley et al., 1996; Friedrich and Korsching, 1997; Ritter et al., 2001; Brustein et al., 2003; Niell and Smith, 2005; Yaksi and Friedrich, 2006; Orger et al., 2008; Ramdya and Engert, 2008; Sumbre et al., 2008; Naumann et al., 2010, 2016; Niessing and Friedrich, 2010; Blumhagen et al., 2011; Zhu et al., 2013; Kubo et al., 2014; Portugues et al., 2014; Candelier et al., 2015; Romano et al., 2015; Pérez-Schuster et al., 2016; Dal Maschio et al., 2017; Pietri et al., 2017) and data analysis (Miri et al., 2011; Freeman et al., 2014) have allowed for the imaging and interpretation of whole brain volumes at high spatial and temporal resolution. Typically, the temporal resolution of these experiments is on the order of few seconds to few miliseconds, enabling to measure neuronal activity with single spike resolution. It is also possible to image the entire brain during free swimming, more close to naturalistic behaviors (Kim et al., 2017). A detailed description of the factors that need to be considered for using two-photon imaging on larval zebrafish have been covered previously (Renninger and Orger, 2013). Instead, we highlight in the following somee important factors that need to be considered for combining functional imaging and EM-based circuit reconstruction.

The acquisition parameters of light microscopes are usually optimized for maximizing temporal resolution and signal-to-noise (SNR) of the activity measurements while minimizing the observable photo damage. At light microscopy level, photo damage is most prominently observable as photo bleaching (Magidson and Khodjakov, 2013). While a comprehensive study of photo damage at ultra structural level in combination with electron microscopy is still missing, several labs and researchers have observed and anecdotally reported that extended LM imaging prior to EM sample preparation can affect the tissue, ultra-structural integrity and staining quality in the subsequent EM steps, even if signs of photo damage are missing on the light microscopy level. It is therefore crucial to reduce photo-damage beyond avoiding photo bleaching. On one hand, this can be achieved by decreasing the laser power under the objective which comes at the cost of sacrificing SNR. On the other hand, decreasing the photon dose by decreasing the dwell time and increasing the imaging rates seems also to reduce photo-toxic effects. The loss in SNR can be compensated partially by using improved transgenic or synthetic reporters.

## 3. Structural imaging

Following functional imaging, the same larvae are prepared for EM. During this process the ultrastructure of the tissue is preserved and stained using a combination of fixatives and heavy metal stains.

### 3.1. Immersion fixation and craniotomy

The tissue fixation is one of the most important steps toward good preservation and staining of cellular ultrastructure. The larval skull consists of soft cartilage covered by connective tissue and skin that hinders the penetration of aldehydes. This layer gets typically removed by a craniotomy. To allow for fast and homogeneous penetration of fixatives such as paraformaldehyde and glutaraldehyde we strongly suggest performing a craniotomy around the brain region of interest as follows (see Wanner et al., 2016b; Vishwanathan et al., 2017 for details on animal procedures):

1. Anesthetize the larva by putting it into a small drop of larval medium (E3 medium) and the anesthetic MS222 (0.1 mg/ml, Sigma E10521).
2. The anesthesia has to be deep enough such that the larva does not show any response/muscle tension to gentle mechanical stimuli such as gentle touches by forceps. Monitor the vital functions of the larva through a stereo microscope. In particular, make sure that there is sufficient blood flow in the brain and monitor the heart beat (~200 beats per minute; Luca et al., 2014).
3. Prepare 2–3% low melting agarose (Sigma A9539) in artificial cerebrospinal fluid (ACSF) and let it cool down to about 35°C . Load a fresh transfer pipette with about 3–4ml of liquid, low melting agarose. Pick up the drop with the larva using the agarose-loaded transfer pipette and mix it well but gentle for 2–3 s.
4. Place the larva with the low melting agarose in a mold and orient the larva using forceps such that you can access the brain region of interest from the top.
5. Let the agarose cure for about 2–5 min.
6. Gently remove any remaining agarose on top of the brain region of interest with a scalpel such that you can easily access the brain to make a craniotomy.
7. Make sure that that region is always covered by ACSF.
8. Now use a sharp-tip tungsten needle or glass-pipette to cut and remove the skin and cartilage generously around the brain region of interest and neighboring areas without damaging the brain. Try not to rip any blood vessels because that can easily cause severe tissue damage.
9. Gently remove the larva from the agarose using a scalpel. Make star like incisions away from the larva and then remove the agarose by pulling it away from the larva to minimize any pressure onto the larva and the exposed brain. Make sure that the exposed brain is always covered by ACSF during this procedure.
10. Make sure that the heart is still beating regularly after the craniotomy.
11. Use a fresh transfer pipette to transfer the larva into freshly prepared fixative at room temperature for 1 h and for 1–23 h in the fridge.

### 3.2. Electron microscopy staining, embedding, and sectioning

**Figure 1:**
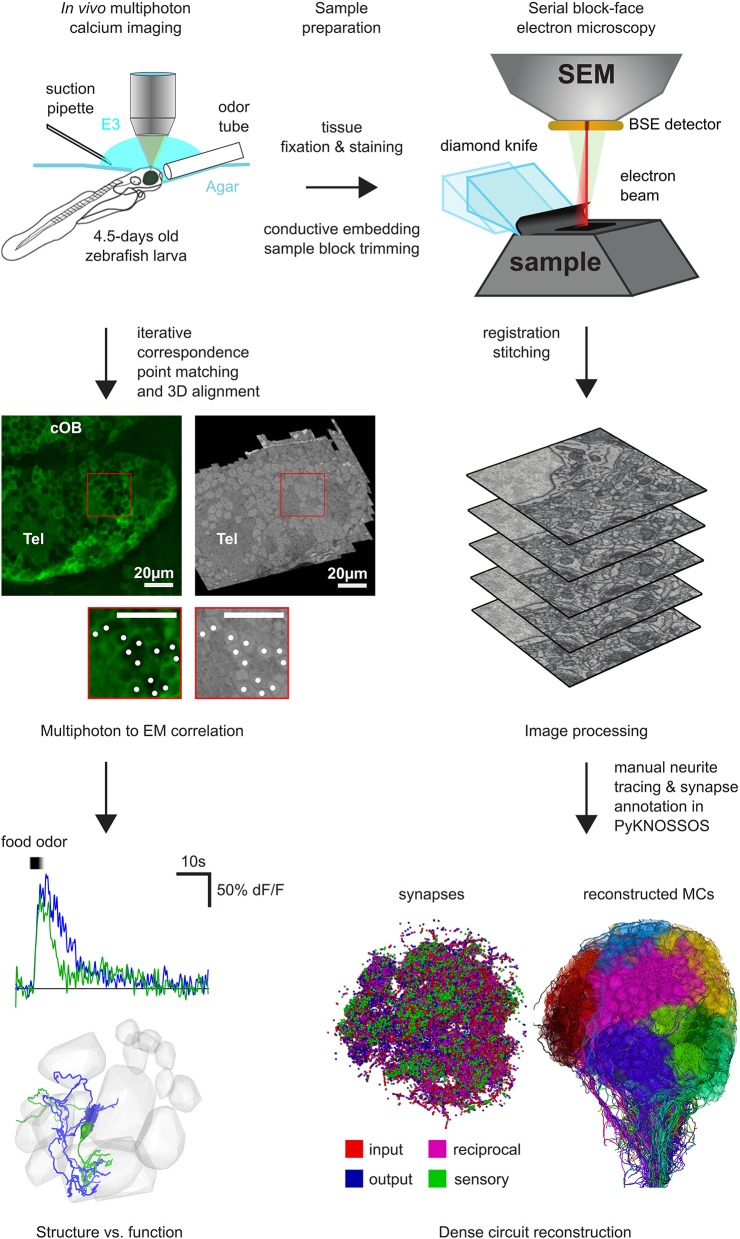
Workflow for SBEM based pipeline: First, two-photon calcium imaging was performed in the OB and the telencephalon over multiple planes to record neuronal activity while delivering different odor stimuli. Next, the same sample was prepared for EM and a complete stack of the OB and parts of the telencephalon was acquired with a SBEM. Subsequently, all neurons in the OB have been reconstructed by manual skeleton tracing (Wanner et al., 2016b). After the co-registration of the EM stack and the two-photon planes, the neuronal activity can be mapped onto the reconstructed neurons for detailed structure to function comparison and analysis.

**Figure 2:**
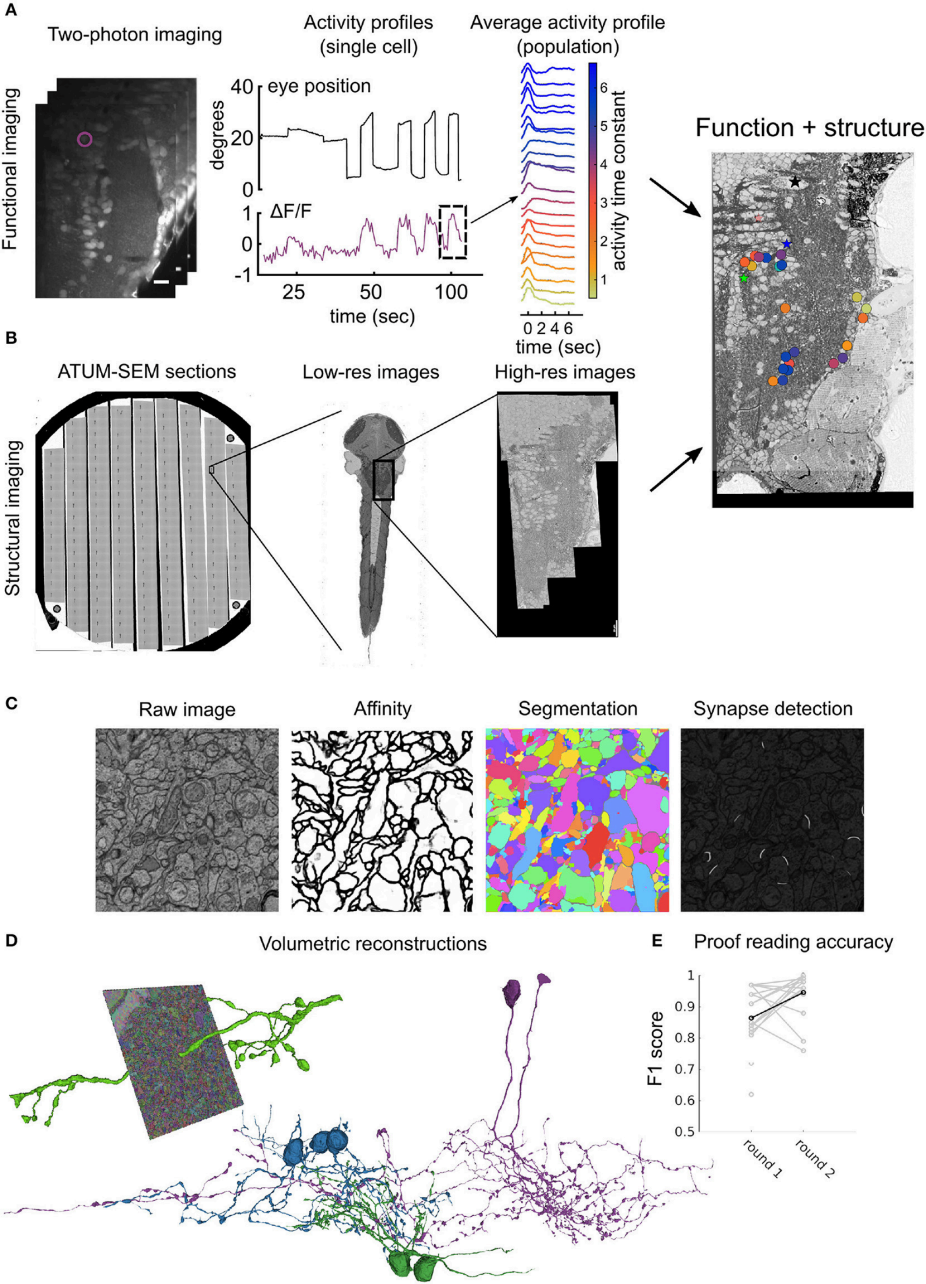
Workflow for ATUM-SEM based pipeline: **(A)** Perform two-photon calcium imaging (left) over the region of interest (in this case hindbrain) over multiple planes to record from neurons while delivering stimulus and/or monitoring behavior (middle). Analyze activity from population offline to compute variable of interest (right) (Vishwanathan et al., 2017). **(B)** Prepare and section the same animal from **(A)** using an ATUM. Prepare sections on conductive substrate (silicon wafer, left) and map all sections in low-resolution (middle) first and then define region corresponding with functionally imaged region for high-resolution imaging (right). After registration of EM images, correspondences between LM and EM are used to register LM images onto EM images to locate somata in both volumes. **(C)** Automatic image segmentation using neural networks is used to generate affinities from raw images, that are then segmented to distinguish neurites from each other. Alternatively, another neural network is used to detect synapses in the entire volume. **(D)** Dense segmentation is agglomerated to produce entire neurites (left). These neurites are proof-read and corrected for mistakes such as false terminations and mergers to reconstruct entire neurons (middle). Colors represent different classes of neurons. **(E)** Accuracy of crowd sourced players reported as F1 scores when proof-reading neurons either the first time (round 1) or the second time (round 2). Each gray dot represents an individual player. Black dot and line is the average.

For heavy metal staining we used conventional reduced Osmium (ROTO) based techniques that impart good contrast to the samples (Deerinck et al., 2010). Briefly, this involves staining with reduced Osmium followed by amplification with thiocarbohydroazine (TCH) and another round of Osmium. This is further amplified by *en bloc* staining of the samples with Uranyl acetate and Lead aspartate (Table 2). We used two different volume EM techniques to acquire large stacks of the zebrafish brain. The olfactory bulb dataset (Figure 1) was acquired with a serial block-face scanning electron microscope (SBEM) (Denk and Horstmann, 2004), whereas the hindbrain dataset (Figure 2) was acquired using automated tape collecting ultra-microtome based scanning electron microscopy (ATUM-SEM) (Schalek et al., 2011). During this process, we have encountered few failure modes as listed in Table 3.

**Table 2:** Fixation and staining comparison.

	**Wanner et al**.	**Vishwanathan et al**.
Fixative	2% Paraformaldehyde, 1% Glutaraldehyde in 0.15 M Cacodylate buffer with 2 mM calcium chloride at pH 7.4. (1h at room temperature, 1 h on ice)	2% Paraformaldehyde, 2.25% Glutaraldehyde in 70 mM Cacodylate buffer at pH 7.4 (over night at 4°C)
Reduced fixation	2% Osmium Tetroxide , 1.5% Potassium Ferrocyanide in 0.15 M Cacodylate buffer with 2 mM calcium chloride (1 h on ice)	1% Osmium Tetroxide , 1.5% Potassium Ferrocyanide in 0.15M Cacodylate buffer (2 h on ice)
Amplification	1% TCH (20min at RT)	1% TCH (15min at RT)
Secondary fixation	2% Osmium tetroxide (30 min at room temperature)	1% Osmium tetroxide (1 h on ice)
Uranyl acetate	1% aqueous UA (overnight at 4°C)	1% aqueous UA (overnight)
Lead aspartate	20 min at 60°C at pH 5.3	30 min at 60°C at pH 5.5
Dehydration in ethanol (in %)	20,50,70,90,100,100 (5 min each)	20,50,70,90,95,2 × 100, 100 - Propylene Oxide (PO) (10 min each)
Resin formulation	11.1 g Glycid ether 6.2 g DDSA 6.25 g MNA Mix very well Add 0.325 ml BDMA Mix and degas	A = 10g LX-112 + 10.9 g NSA ; B = 18 g LX-112+ 15.5 g NSA; 3A+7B+2%BDMA.

**Table 3:** Failure modes.

	**Serial block-face scanning electron microscopy (SBEM) (Wanner et al., 2016b)**	**Automated tape collecting ultra-microtome scanning electron microscopy (ATUM-SEM) (Vishwanathan et al., 2017)**
Tissue integrity	1. Dissociated, dense tissue and broken or jagged membranes indicate poor tissue fixation. This can be addressed by increasing the size of the craniotomy and/or moving the craniotomy closer to the region of interest.	
	2. Broken or jagged membranes and exploded mitochondria may indicate problems with the osmolarity of the ACSF.	
	3. Cracks in the tissue may indicate problems with dehydration.	
Tissue staining	1. Bands of precipitates of the stains are occasionally observed in the neuropil. This can be avoided by having clean large cranial access and longer wash times.	
	2. Low contrast can indicate that the pH of the lead aspartate was not within the optimal range of 5.3–5.7 or that the craniotomy was not large enough.	
Tissue sectioning problems	1. For reliable 25–30 nm thin sectioning on the SBEM it is important carefully trim the sample to a rectangular pyramid with smooth faces, usually falling of at an angle of 46–48 degree. 2. Multiple beam exposure of the same area can impair reliability and quality of the cutting. To cover larger FOV, use a mosaic of tiles with alternating overlap to avoid having regions that are scanned four times. 3. For reliable 25–30 nm thin sectioning, it is crucial to use a fresh knife and to keep the electron dose and energy to a minimum. Typical parameter settings result in an electron dose of about 14–18 electrons per nm^2^ and landing energies of < 2 keV.	1. Reliable series collection requires an accurate mesa (rectangular profile was used) and preferably a new knife for cutting. 2. Folds observed at the interface of tissue and resin. This can be overcome by using a resin formulation that has low viscosity during infiltration. 3. Another source of folds can be caused by hydrophobic tape substrate. This can be avoided by glow discharging the substrate prior to collection.
Tissue imaging problems	Use conductive embedding procedures such as E/E embedding (Wanner et al., 2016b) or adding carbon black to the resin (Nguyen et al., 2016) to reduce charging artifacts.	1. Charging can sometime occur for very thin layers of evaporated Carbon. This can be avoided if >5 nm of Carbon is coated. Poor contrast in sections can be enhanced by post staining the sections. 2. Charging can also be avoided by collecting sections on conductive substrates (Kubota et al., 2018).

During the staining of the tissue we observed poor penetration or precipitation of the stain in the form of large contrast gradients in the images. In both cases, beginning with good craniotomies was able to mitigate these problems. Occasionally, reducing the amount of TCH helped to reduce the occurrence of precipitates. Depending on the acquisition method, different resins should be used for embedding. In SBEM, surface charging due to electrons that accumulate in regions with low conductivity/heavy metal content is a common problem (Wanner et al., 2015; Titze and Genoud, 2016). Besides the image saturation due to accumulating electrons, the charging can impair the cutting quality and even more importantly, it can lead to non-linear, non-stationary distortions in the images, which can complicate the stitching of a mosaic of overlapping tiles tremendously. These effects are in particular problematic for zebrafish samples, because there is typically empty resin surrounding the brain tissue. One way to cope with this problem is to use variable pressure SEM (Griffin, 2007). This technique reduces charging artifacts by adding a gaseous agent into the recording chamber (e.g., water or nitrogen) whose molecules get ionized and neutralize excessive electrons on the block surface. These agents typically compromise the vacuum in the chamber and scatter electrons which can severely affect the resolution and SNR in the images. However, a promising new focal gas injection-based charge compensation seems to largely mitigate the charging problems without compromising the SNR (Deerinck et al., 2017). Another, technically more challenging method is to introduce a sputter-coating device into the recording chamber that coats the sample surface after each cut with a thin layer of Chromium or Palladium and makes its surface perfectly conductive (Titze and Denk, 2013). An alternative approach is to improve the grounding of the tissue and the sample block by adding conductive material such as carbon black (Nguyen et al., 2016) into the otherwise empty resin space surrounding the tissue. In the case of the larval OB image stack (Wanner et al., 2016b) an alternative embedding method called Epo-tek and Epon (EE) embedding was developed, in which the tissue was surrounded with a silver-filled epoxy glue before curing the Epon. EE-embedding effectively resolves the surface charging problems during backscattered electron imaging and is therefore suitable for blockface imaging in high vacuum mode. This also results in an order of magnitude increase in both, SNR and acquisition speed (Wanner et al., 2016b). In the following we give a step-by-step description of the procedure for EE-embedding of a resin-immersed tissue sample:

1. Normal sample preparation (fixation, staining, dehydration, etc.).
2. Immerse sample in resin (Epon in this case) for 4h to overnight.
3. Prepare a small batch of EPO-TEK® EE129-4 compound A and B with ratio A:B = 1.25:1. Typically, we use 0.5g A and 0.4g B for 2 zebrafish larvae. Perform the following steps quickly (within a couple of minutes), because the conductive glue becomes more viscous over time.
4. Mix compounds A & B very well with a toothpick.
5. Fill the mixed conductive glue carefully into a mold. Make sure that there are no air bubbles. Vacuum degassing might help.
6. Take the sample out of the resin, for example by using a toothpick such that the larva sticks to the tip of the toothpick.
7. Remove remaining resin around the sample using gravity or by carefully wiping the sample surface with a tissue.
8. Put the sample into the mold with the conductive glue. Make sure that there is as little resin as possible getting into the mold.
9. Mix the sample very well and very carefully with the conductive glue. Because the conductive glue is opaque it can be useful to only immerse the parts of the sample that are going to be imaged in the conductive glue. The rest (e.g., the larval tail) can be used to gently move the sample around (tilting and rotating) with a toothpick in order to mix it with the conductive support.
10. Cure the embedded samples in a 60°C oven for 48 h.

For ATUM-SEM, since the sample is collected prior to imaging, the resin had to be customized in order to facilitate good cutting characteristics. Embedding the samples in most typical resins resulted in the formation of micro-folds and compression of the tissue at the tissue-resin interface. This kind of folds can typically be attributed to the change in the density at the interface between the tissue and the resin. In order to overcome these problems, one approach is to re-embed the sample with resin that has been made more dense by the addition of tissue slurry that acts to homogenize the resin. Another similar approach was to embed the larvae inside a larger piece of tissue that then serves to homogenize the resin (Hildebrand et al., 2017). Our approach was to design a low-viscosity resin, that was able to withstand the compression at the interface while retaining good cutting characteristics (Table 2). This resin allowed for collection of 1000's of fold free sections from zebrafish larvae and murine tissue.

## 4. Neuron reconstruction

We have employed two different methods for neuron reconstruction. In the case of the larval OB, neurons were skeletonized manually by a cohort of more than 30 professional image annotators (Wanner et al., 2016b), whereas in the case of the hindbrain, crowd-sourced players and professional image annotators proofread an automated, volumetric reconstruction (Kim et al., 2014). There are pros and cons to each of these methods, and here we list some of them, based on our experiences.

### 4.1. Skeleton based reconstructions

Despite the fact that automated image segmentation methods have made tremendous progress in the last few years, manual neuron reconstruction is still the preferred and often more economical approach for small and intermediate sized reconstruction projects involving few hundreds to few thousands of neurons. While manual volumetric annotation is extremely time consuming, skeleton tracing of neurites usually is sufficient for many circuit neuroscience related questions and is orders of magnitudes faster (Helmstaedter et al., 2011). Neurons are traced manually by placing connected nodes onto cross-sections of neurites in the image data, many such nodes are then connected to form entire neurons. This is typically done using open-sourced software packages such as Catmaid (Saalfeld et al., 2009), KNOSSOS (Helmstaedter et al., 2011), and PyKNOSSOS (Wanner et al., 2016a). These software tools are specifically designed for high-throughput, multi-user, 3D image annotation and neuron reconstruction. Typically, skeleton tracing is performed by cohorts of students or researchers. A motivated researcher or student can learn a lot about the underlying data while manually annotating neurons, but it is probably not the best use of their talents to trace neurons for several thousands of hours (Helmstaedter et al., 2011). However, crowd sourcing neuron reconstruction and synapse annotation is intrinsically difficult. First, tracing neurons is not trivial and it requires 10–40 h of training for a naive student to become a good annotator (Helmstaedter et al., 2011). Second, neuron tracing is relatively monotonous and only few people are willing to do this kind of work over a prolonged period of time with the necessary care and accuracy. Helmstaedter et al. developed a redundant-skeleton consensus procedure (RESCOP) that can be used for reliable neuron reconstruction with cohorts of weakly trained students. RESCOP was used to densely reconstruct 950 neurons in the inner plexiform layer of a mouse retina (Helmstaedter et al., 2013). However, manual tracing is an error-prone process, even if performed by expert annotators. A single expert annotator misses on average at least 10 percent of the true neuronal arbor (Wanner et al., 2016b). Therefore, expert revision and/or redundant annotation is typically used to leverage the accuracy of the resulting reconstruction (Helmstaedter et al., 2011; Schneider-Mizell et al., 2016; Wanner et al., 2016b). In the case of the mouse inner plexiform layer connectome, RESCOP required an average redundancy of 6 for ganglion cells and 4 for amacrine and bipolar cells.

While tracing straight neurites is typically faster than tracing branching neurites with complex morphology, the tracing speed of a single annotator is on average 2–15 h per mm neurite length (Helmstaedter et al., 2011; Wanner et al., 2016b; Boergens et al., 2017). Hence, redundant reconstruction can be time consuming and costly. Therefore a new iterative consensus procedure called CORE (“COnvergence by Redundancy and Experts”) (Wanner et al., 2016b) was developed to reconstruct >1,000 neurons in the larval zebrafish OB. CORE leverages redundant reconstruction with focused expert input. For the OB reconstruction CORE achieved very high accuracy (F1 score >0.989 for mitral cells) with just a three-fold redundant reconstruction together with local re-tracing at mismatch points and focused expert inspection. Thereby the bulk skeleton tracing was outsourced to professional annotators (www.ariadne.ai).

### 4.2. Segmentation based volumetric reconstructions

Volumetric reconstructions generally mean “coloring” entire neurons, including intracellular regions. In contrast to skeletons, this method effectively captures detailed morphologies of the neurons, including spine architecture and gives an accurate representation of the changes in the thickness of the neurites that originate from the somata. Manual volume annotation, although accurate, is very laborious, time consuming and about 50 times slower than skeletonization (Helmstaedter et al., 2011). Recent advances in machine learning tools such as deep convolutional networks (CNNs) have been developed to segment entire images based on human generated ground-truth annotations (Chklovskii et al., 2010; Jain et al., 2010; Kreshuk et al., 2011; Andres et al., 2012; Berning et al., 2015; Kaynig et al., 2015; Lee et al., 2015; Dorkenwald et al., 2017; Staffler et al., 2017). Typically the process requires (Figure 2):

1. Accurate painting of all the objects, neurites and boundaries also referred to as the ground-truth annotation.
2. Training of a neural net to recognize and classify pixels as belonging to a boundary or not.
3. Segmenting neurites based on this boundary detection.
4. Agglomerating segments to reconstruct entire neurons.

Similar approaches can be employed for other features of interest, for example neural networks can be trained to identify synapses, mitochondria etc. Using the above described methods, we have automatically segmented the hindbrain dataset in order to reconstruct entire volumetric profiles of neurons. To validate and to correct the mistakes that are made by these machine learning algorithms, we use a crowd-sourcing platform where experienced players check the validity of the algorithms and override in regions where the AI makes mistakes (Kim et al., 2014). The typical workflow for a single neuron requires:

1. Seeding of the neuron of interest.
2. Letting the AI populate the entire neuron.
3. Human proofreading of false terminations and mergers
4. Correcting identified mistakes.

To ensure high accuracy, this process is performed twice in a “wikipedia” like manner, where the first player proof-reads and checks for errors in round 1 and a second player then checks that intermediate result in round 2 with the latest player having veto privileges. Finally, the entire neuron is checked by experts, who have >5,000 h of expertise to mark the neurons as complete. Using this process on average we can accurately reconstruct 3–4 neurons per day, with an average of 1.6 mm neurite length per day with a coverage factor of 3, which means each neuron had been reconstructed by 2 players and proof read by 1 expert. The crowd sourced players have F1 scores on average >0.8 as compared to expert tracers.

## 5. Correlation of function to structure

A long standing question in neuroscience is whether and how the structure of neuronal circuits determines their function. A directly related question is to what extent knowledge about circuit structure can predict circuit function (Lichtman and Sanes, 2008; Seung, 2009; Bargmann, 2012; Morgan and Lichtman, 2013). Although fundamental, these questions remain unresolved for many circuits, largely because the detailed analysis of circuit structure, or connectivity, is still a major challenge. The first step involves mapping the neuronal activity from calcium imaging to the reconstructed neurons from EM based circuit reconstruction. To do this it is necessary to precisely register the calcium imaging planes to the electron microscopy image stack. Typically, this is done by iterative point matching and 3D alignment between the LM and EM data. Corresponding landmarks such as prominent blood vessel patterns or unique soma locations can be identified in both datasets. These landmarks can be used to calculate a spatial transform between the LM and the EM data. Tools for performing point matching are available on open sourced platforms (Table 1—software) and can be easily scripted using built-in functions in Python (www.python.org) and Matlab (www.mathworks.com). The reconstructed connectivity or wiring diagrams can be used for hypothesis testing of circuit models. Using two recent larval zebrafish circuit reconstruction studies, we provide two examples of hypotheses that can be tested from such connectomes.

**Example 1:** In the hindbrain, eye position encoding neurons persistently fire action potentials during eye fixations (McFarland and Fuchs, 1992; Aksay et al., 2000). These neurons transform eye velocity signals to eye position signals and are called velocity-to-position-integrator (VPNI) neurons. Theoretical models suggest that persistent activity could be induced by recurrent connectivity between VPNI neurons (Cannon et al., 1983; Seung, 1996). To test this hypothesis we reconstructed the connectivity between functionally identified VPNI neurons (Vishwanathan et al., 2017). From these reconstructions we found that the VPNI neurons are not a homogeneous class of neurons. We observed at least three classes of neurons, two excitatory and one inhibitory, that differed in their morphology, synaptic distribution and axonal targets. We further observed that only the excitatory class of neurons were recurrently connected, which supports the idea of positive feedback as one of the mechanisms by which persistent activity can be implemented.

**Example 2:** In the OB chemically similar odors tend to activate overlapping sets of olfactory glomeruli. This activity is decorrelated and normalized, presumably by interactions between interneurons (INs) and mitral cells (MCs), the major output neurons of the OB (Friedrich and Laurent, 2001; Yaksi et al., 2007; Niessing and Friedrich, 2010; Zhu et al., 2013). However, a mechanistic understanding of these population-level computations is lacking. By the dense reconstruction of all OB neurons we found that most MCs are largely uniglomerular (Wanner et al., 2016b). In contrast, INs tend to innervate multiple glomeruli and the glomerular IN innervation is governed by glomerular identity. Moreover, the examined INs did not have specific input or output glomeruli, implying that interglomerular interactions have a strong non-directional component. As a consequence, selective interglomerular connectivity may support differential preprocessing of odor information that is routed to specific target regions and that is relevant for different behaviors. Moreover, the specific projections between glomeruli may favor inhibitory interactions between processing channels with specific tuning properties which in turn could be an efficient solution for decorrelating activity patterns between small groups of neurons. This kind of questions can only be tested with experiments in which both, connectivity and activity, are measured exhaustively with single neuron resolution.

## 6. Conclusions

Detailed anatomical maps and wiring diagrams can be a very powerful tool not only for gaining a mechanistic understanding of brain function, but perhaps even more importantly as a source of inspiration for new models and hypotheses for circuit functions. Here, we presented some of the tools, methods and examples that are required for large scale circuit reconstruction, based on our work in the larval zebrafish. We hope that this article helps lowering the threshold for combining synaptic resolution circuit reconstruction and functional imaging. We highlighted two different sets of methods that were used to study the larval zebrafish. Both highlighted methods have advantages and disadvantages that the end user should consider before embarking on similar studies. Other important factors that have to be considered for large-scale volume EM projects, such as image acquisition speed, have been extensively discussed in previous reviews (Briggman and Bock, 2012; Wanner et al., 2015). Many of the tools that were used in the studies presented here are available in the form of open sourced software (Table 1) with more tools becoming available every day, ultimately making it possible to routinely analyze wiring diagrams.

## Ethics statement

All zebrafish larvae experimental procedures for the hindbrain dataset were approved by Weill Cornell Medical College's Institutional Animal Care and Use Committee. All animal procedures for the olfactory bulb dataset were approved by the Veterinary Department of the Canton Basel-Stadt (Switzerland).

## Author contributions

AV acquired and analyzed the larval zebrafish hind brain dataset and wrote the manuscript. AW acquired and analyzed the larval zebrafish olfactory bulb dataset and wrote the manuscript.

### Conflict of interest statement

Part of the results disclosed herein have been included in European patent application EP14736451 and US patent application US14897514. AW is owner and CEO of Ariadne-Service Gmbh.

The remaining author declares that the research was conducted in the absence of any commercial or financial relationships that could be construed as a potential conflict of interest.
